# Human D‐Lactate Dehydrogenase Deficiency: A Case Report in a Young Boy

**DOI:** 10.1002/jmd2.70039

**Published:** 2025-07-17

**Authors:** T. B. Sloth, M. C. Ørngreen, J. Ek, I. Bache, F. Wibrand, A. M. Lund

**Affiliations:** ^1^ Centre of Inherited Metabolic Diseases, Department of Pediatric and Adolescent Medicine Rigshospitalet, Copenhagen University Hospital Copenhagen Denmark; ^2^ Department of Clinical Genetics Rigshospitalet, Copenhagen University Hospital Copenhagen Denmark; ^3^ Department of Clinical Medicine, Faculty of Health and Medical Sciences University of Copenhagen Copenhagen Denmark

**Keywords:** developmental delay, D‐lactate, genetics, gout, human D‐lactate dehydrogenase

## Abstract

D‐lactate is an isomeric form of lactate, which is almost undetectable in the circulation in individuals with normal lactate metabolism. Patients diagnosed with the disease human D‐lactate dehydrogenase deficiency present with elevated plasma D‐lactate, causing D‐lactic acidosis, which can be associated with neurological symptoms. This paper reports a Danish patient presenting with delayed psycho‐motor development and metabolic acidosis. Whole genome sequencing (WGS) revealed a homozygous 0.1 Mb loss of the long arm of chromosome 16 involving 3 protein coding genes, *CTRB2*, *ZFP1*, and exon 1–7 of the *LDHD* gene (NM_194436.3c: 1_930del, p.M1_Q310del), which encodes the human D‐lactate dehydrogenase enzyme. Metabolic screening of the plasma and urine demonstrated elevated levels of D‐lactate. Based on the genetic and biochemical findings, the patient was diagnosed with human D‐lactate dehydrogenase deficiency. Biochemical and molecular studies, including WGS, did not disclose any additional pathogenic findings. This report compares the laboratory and clinical findings in our patient with observations in other patients diagnosed with human D‐lactate dehydrogenase deficiency described in the literature. The comparison shows that the clinical symptoms vary among patients with pathogenic variants in the *LDHD* gene, but an elevated level of D‐lactate in plasma and urine is found in all patients. Some reported patients had additional diseases, while the boy reported here was most probably solely affected by D‐lactate dehydrogenase deficiency, making the clinical picture clearer. Take‐home message: Human D‐lactate dehydrogenase deficiency can be caused by pathogenic variants of the *LDHD* gene, and shows a broad phenotypic variability, with some patients only having increased plasma urate/gout and others neurological findings and psycho‐motor developmental delay.

**Summary**

- Pathogenic variants of the *LDHD* gene can lead to D‐lactate acidosis.
- D‐lactate acidosis can be associated with psycho‐motor developmental delay.
- Human LDHD deficiency has a broad phenotypic variability, with some patients only having increased plasma urate/gout and others having neurological findings and psycho‐motor developmental delay.

## 1 Introduction

Lactate is a naturally occurring organic acid found in the human body in two isomeric forms, L‐ and D‐lactate. The two enantiomers differ by a chiral C2 atom [1]. In individuals with normal lactate metabolism, L‐lactate is detectable in plasma in the mmol range, and the concentration is approximately 100 times higher than the amount of D‐lactate [2]. In humans, L‐lactate is metabolized by NAD^+^‐dependent L‐lactate dehydrogenase [3]. D‐lactate can originate from exogenous sources like food products as well as endogenously via the methylglyoxal metabolic pathway [1, 4, 5, 6]. The latter converts the toxic methylglyoxal into D‐lactate, which is not toxic in small amounts. The formed or absorbed D‐lactate can be oxidized, catalyzed by the enzyme D‐lactate dehydrogenase (EC: 1.1.2.4 LDHD), into pyruvate [1].

Elimination of lactate is essential to prevent accumulation of lactate and thus lactic acidosis. In most cases, lactic acidosis is due to an increased amount of L‐lactate [7] while accumulation of D‐lactate is a rare condition [4]. D‐lactate acidosis with neurological symptoms is also referred to as D‐lactate encephalopathy, underlining that D‐lactate may have a neurological toxic effect [4, 8]. Both isomeric forms of lactate can pass the blood–brain barrier, but the toxic effect on the brain is only attributed to D‐lactate [1, 9].

D‐lactic acidosis is well known in connection with resection of the small intestine (short‐bowel syndrome) [5, 10], but rarely do patients have increased levels of D‐lactate in plasma without prior bowel resection [2]. The increased concentration of D‐lactate in these patients may be caused by a lack of LDHD activity, which is the case in the disease human D‐lactate dehydrogenase deficiency (OMIM 245450). This disease is caused by pathogenic variants in the *LDHD* gene (OMIM 607490) leading to decreased activity of LDHD. Human D‐lactate dehydrogenase deficiency has only been described in a few patients globally [2, 4, 11, 12]. In some of the patients, early clinical presentation with delayed mental development has been described [4, 11]; in most patients with delayed development, the clinical pictures have been confounded by the co‐existence of other pathologies like 11p deletion syndrome [2, 4]. A Danish patient with delayed mental development was diagnosed with human D‐lactate dehydrogenase deficiency with no documentation of co‐existing diseases and is described in this report.

## 2 Methods

Clinical data were obtained retrospectively from electronic patient records. The family gave consent before any data were collected. Some data cannot be presented here because of the person‐sensitive nature of the data but may be detailed upon reasonable request.

Data from literature were found through a search in PubMed on the 30th of March 2024 using the following search words (“acidosis, lactic” OR “lactic acidosis” OR “lactate acidosis” OR “hyperlactatemia” OR “hyperuricemia” OR “D‐lactate acidosis” OR “D‐lactic acidosis”) AND (“lactate dehydrogenase/genetics” OR “D‐ lactate dehydrogenase deficiency” OR “human D‐lactate dehydrogenase deficiency” OR “D‐LDH deficiency” OR “LDHD deficiency” OR “D‐lactate acidosis syndrome” OR “LDHD mutation”).

Assays for routine clinical chemistry, biochemical genetics, and molecular genetics were performed using methods that can be described upon request. Determinations of D‐lactate in urine and plasma are not routine assays and were performed using a D‐lactate assay kit (Cayman Chemical).

## 3 Case Presentation

This Danish boy was born at term after an uneventful pregnancy and delivery. The patient's parents are from the same village in Iraq and distantly related.

The patient was referred to us at the age of 3 years with mild to moderate psycho‐motor developmental delay and metabolic acidosis. His motor function was not age‐appropriate; his running was uncoordinated, and he had a wide‐based gait with uncoordinated movements of his arms. Additionally, he had pes planus and valgus positioning of the ankles bilaterally. The reflexes were difficult to trigger, and he was generally hypotonic. He was delayed both expressively and in comprehension, using only a few words and struggling to understand and follow instructions. A neuropsychological test showed that the patient had a reduced level at about half his chronological age of functioning compared to individuals at the same age. Of note, the patient's home is bilingual, but the extent of his delay cannot be explained by that. A psychiatric assessment revealed reduced eye contact, facial expressions, and gestures. He was given the provisional diagnosis of Pervasive Developmental Disorder, Unspecified (ICD‐10 code 84.9). The patient is expected to be further assessed within the autism spectrum at a later age.

By objective examination, a solitary café‐au‐lait spot was found on his truncus. He was not dysmorphic. Ultrasound examination of the abdomen showed a normal urinary tract and liver with no signs of hepatosplenomegaly. Echocardiography was normal.

Routine paraclinical investigations showed normal hematology, electrolytes, lipid profile, and kidney and liver function. Urate was slightly elevated at 0.38 (mmol/L; ref: 0.12–0.32). Acid–base balance showed decreased pH at 7.24, base excess at −4.8 mmol/L, and reduced standard‐bicarbonate at 19.5 mmol/L; two measurements of p‐L‐lactate were normal at 0.9 and 1.5 mmol/L, respectively.

An array‐CGH showed homozygosity for an approximate 0.1 Mb loss of the long arm of chromosome 16: arr[GRCh37] 16q23.1 (75149945_75,238 039) × 0. The loss involved the first exons of the *LDHD* gene in addition to *ZFP1* (zinc finger protein, OMIM # 617230) and *CTRB2* (Chymotrypsinogen B2, OMIM #619620). There is no known disease association with *ZFP1* nor *CTRB2* and no loss of function models generated in other species for these two genes. Since there is a lack of knowledge about *ZFP1* and *CTRB2*, we cannot draw any conclusions about their significance. Consequently, it cannot formally be ruled out that one or both genes contribute to the patient's neurological phenotype. Trio‐WGS was performed, primarily confirming and further defining the homozygotic loss of a 0.1 Mb deletion seq[GRCh38]16q23.1 (75113770–75 209 506) × 0 involving exon one to seven of the *LDHD* gene (NM_194436.3c: 1_930del, p.M1_Q310del). Analysis of the parents' genetic material confirmed heterozygosity for the same deletion in both parents. To rule out other genetic conditions contributing to the phenotype, filtering and interpretation of variants (small nucleotides variants (snv's) and structural variation (SV)) were performed. Briefly, variants with a minor allele frequency > 0.01 in gnomAD or in an in‐house frequency database were removed. Silent, intergenic, and deep intronic variants not predicted to affect mRNA splicing were removed. Finally, only variants affecting genes associated with disease (OMIM, HGMD, Genomic England panelapp) were retained. Further, data from WGS using number of snv's measuring mother/father kinship‐coefficient and heterozygous/homozygous ratios shows consanguinity corresponding to parents being distant cousins. Thus, the patient was genetically diagnosed with human D‐lactate dehydrogenase deficiency, which was also supported by the metabolic findings in the urine and plasma.

Metabolic analysis showed normal amino acids and acylcarnitines in plasma. A urine metabolic screening showed elevated excretion of 2‐hydroxyisovaleric acid and 2‐hydroxyisocaproic acid, as well as traces of 2‐hydroxy‐3‐methylvaleric acid and very increased excretion of lactate. This analysis of organic acids does not differentiate between D‐ and L‐isomers. Analysis of D‐lactate using a D‐lactate assay showed elevated D‐lactate at 1.4 mmol/mol creatinine (ref: < 0.021 mmol/mol creatinine). P‐D‐lactate was elevated at 1.2 mmol/L (ref: < 0.026 mmol/L). Values are shown in Table 1. The pattern of excretion of the mentioned organic acids is characteristic for conditions with increased excretion of D‐lactate and has been found in other patients with D‐lactate acidosis [1, 2].

**TABLE 1:** Laboratory data in Danish boy with LDHD deficiency.

	Result	Reference
D‐lactate in urine	1.4 mmol/mol creatinine	< 0.021 mmol/mol creatinine
D‐lactate in plasma	1.2 mmol/L	< 0.026 mmol/L
Lactate in urine	Very increased (qualitatively)	—
2‐hydroxyisovaleric acid in urine	Elevated (qualitatively)	—
2‐hydroxyisocaproic acid in urine	Elevated (qualitatively)	—
2‐hydroxy‐3‐methylvaleric acid in urine	Detected (qualitatively)	—
Base excess	−4.8 mmol/L	−2 – 2 mmol/L
Plasma pH	7.24	7.35–7.45
Plasma Urate	0.38 mmol/L	0.12–0.32 mmol/L
Plasma L‐lactate	0.9 mmol/L	0.5–1.8 mmol/L
Measured twice	1.5 mmol/L	

Besides our patient, pathogenic variants in the *LDHD* gene have been identified in 18 other patients (from seven families) described in other reports. Table 2 provides a summary of the clinical, biochemical, and genetic findings in these patients [2, 4, 11, 12, 13].

**TABLE 2:** Overview of patients identified with LDHD deficiency.

	Drabkin et al. [12]	Bardin et al. [11]	Bardin et al. [11]	Bardin et al. [11]	Monroe et al. [2], Duran et al. [13]	Monroe et al. [2]	Kwong et al. [4]	Our patient
Family	Bedouin‐Israeli kindred. Consanguineous	Melanesian family, three affected siblings. Non‐consanguineous	Vietnamese family, two affected siblings. Non‐consanguineous	Parents from Algeria Consanguinity not mentioned	Parents from the same Sicilian village in Italy. Share some degree of consanguinity	Parents from Moluccas, Indonesian Consanguineous	Parents from China. Non‐consanguineous	Parents from the same Iraqi village Share some degree of consanguinity
Mental and motor development	No neurological symptoms reported	No neurological symptoms reported	One of the patients developed severe neurological features at the age of 34 years, but was normal earlier in life No neurological symptoms reported in the other patient	No neurological symptoms reported	Severe delay in mental development, delay in motor development, behavioral problems, and intellectual disability	Delayed motor and mental development, lost social interaction, severe hypotonia and epilepsy until the age of 14 years The patient was clinically diagnosed with West Syndrome	Delayed mental development evolving into limited intelligence, mild transient ataxia, and mild central hypotonia	Delayed motor and mental development including mild hypotonia, abnormal walking and running style, and delay expressively and in comprehension The patient was diagnosed with Pervasive Developmental Disorder, Unspecified (ICD‐10 code 84.9)
Other clinical features	Gout arthropathy in both adults and children	Early‐onset gout Started at age 13, 16, and 21 years, respectively	Early‐onset gout Started at age 21 and 9 years, respectively	Early‐onset gout Started at age 19 years	Cryptorchidism, blindness (aniridia, with later onset of cataract and glaucoma), slanting eyelids, protruding lower lip, and mildly dysplastic helices	No other clinical features found	Mild transient hepatomegaly in infancy	Solitary café‐au‐lait spot
D‐lactate	Elevated average plasma D‐lactate: 3.16 ± 0.63 mmol/L (ref: in healthy controls: undetectable, normal levels: < 0.043 mmol/L) Elevated average D‐lactate in urine: 32.08 ± 5.77 mmol/L (ref: in healthy controls: 0.65 mmol/L ± 0.65 mmol/L, normal levels: 0.0–0.25 mmol/L)	Serum D‐lactate: 0.306 mmol/L, 0.558 mmol/L, and 0.632 mmol/L Elevated D‐lactate in urine: 21.5 mmol/L, 7.8 mmol/L, and 18.1 mmol/L	Not measured	Plasma D‐lactate: > 0.5 mmol/L Elevated D‐lactate in urine: 6.15 mmol/L	Plasma D‐lactate: 0.7 mmol/L Elevated D‐lactate excretion at 1686 mmol/mol creatinine	Plasma D‐lactate: 1.1–1.2 mmol/L Elevated D‐lactate excretion	Serum D‐lactate: 0.61 mmol/L (ref: 0–0.25 mmol/L)	Plasma D‐lactate: 1.2 mmol/L (ref: < 0.026 mmol/L, *n* = 10) Elevated D‐lactate in urine: 1.4 mmol/mol creatinine (ref: < 0.021 mmol/mol creatinine, *n* = 10)
Other D‐2‐hydroxyacids	Not mentioned	Not mentioned	Not mentioned	Not mentioned	Elevated 2‐hydroxyisovaleric acid and 2‐hydroxyisocaproic acid in plasma and urine	Elevated 2‐hydroxyisovaleric acid and 2‐hydroxyisocaproic acid in plasma and urine	Elevated 3‐hydroxybutyric acid, 2‐hydroxyisovaleric acid and 2‐hydroxy‐3‐methylvaleric acid, and 2‐hydroxy‐isocaproic acid in urine	Elevated 2‐hydroxyisovaleric acid and 2‐hydroxyisocaproic acid, as well as traces of 2‐hydroxy‐3‐methylvaleric acid in urine
Uric acid	Elevated average plasma uric acid: Adults: 10.34 mg/dL ± 1.84 mg/dL (normal levels: 3.5–7.2 mg/dL) Children: 6.75 mg/dL ± 0.7 mg/dL (normal levels: 2–6.2 mg/dL) Uric acid in four affected individuals: 24.32 ± 15.3 mg/dL (normal levels: 37–92 mg/dL)	Elevated serum uric acid at 0.71, 0.74, and 0.94 mmol/L, respectively	Elevated serum uric acid at 0.55 and 0.58 mmol/L, respectively	Elevated serum uric acid at 0.46 mmol/L (ref: 0.24 mmol/L—0.51 mmol/L)	Not mentioned	Not mentioned	Plasma uric acid levels at 0.32 mmol/L (normal range: 0.11–0.3 mmol/L)	Elevated plasma uric acid at 0.38 mmol/L (ref: 0.12 mmol/L—0.32 mmol/L)
LDHD variants identified	Homozygous missense variant c.1108C>T, p. (Arg370Trp) (NM_153486.3)	Homozygous variant c.206 T>C; rs1035398551 p.(Val69Ala) (NM_153486.3)	Homozygous nonsense variant c.1363dupG, p. (AlaGly432fsTer58) (NM_153486.3)	Homozygous missense variant c.752C>T, p. (Thr251Met) (NM_153486.3)	Homozygous missense variant c.1388C>T, p. (Thr463Met) (NM_153486.3)	Homozygous missense variant c.1122G>T, p. (Trp374Cys) (NM_153486.3)	Compound heterozygous splice site variant c.469 + 1dupG, (NM_153486.3) and missense variant c.752C>T, p. (Thr251Met) (NM_153486.3)	Homozygous deletion, p.M1_Q310del (NM_194436.3)
Other genetic variants	No other genetic variants identified	No other genetic variants described	Homozygous undescribed frameshift variant of the *RHBG* gene c.1064dupT, p. (Val357GlyfsTer10) (NM_020407.5)	No other genetic variants identified	De novo 11p13 deletion (11p deletion syndrome)	Heterozygous de novo variant in *CACNA1B* c.1429C>T, p. (Arg477Cys) (NM_000718.3), thought to explain West syndrome	No other genetic variants identified, but mitochondrial complex IV deficiency was found	No other genetic variants identified

Four of the patients, including our patient, have consanguineous parents, and all patients, except one, have homozygous pathogenic variants in the *LDHD* gene of varying types. In five patients, genetic variants in genes other than the *LDHD* gene have been observed, making it difficult to know which clinical features are caused by the *LDHD* variants. The metabolic profile in these patients correlates well with what we have found in our patient.

## 4 Discussion

LDHD catalyzes the oxidation of D‐lactate to pyruvate. Previously, this conversion has been attributed to D‐2‐hydroxyacid dehydrogenase [2]. LDHD is categorized based on the co‐factor dependency; namely FAD‐ and NAD‐dependent LDHDs. The FAD‐dependent LDHD was first identified in other organisms, but has later been found in mammals, including humans and mice. FAD‐dependent LDHD is a member of the VAO/PCM flavo protein family [1, 14].

D‐lactate and other D‐2‐hydroxyacids, including D‐2‐hydroxyisovalerate, D‐2‐hydroxyisocaproate, and 2‐hydroxy‐3‐methylvalerate, contain a D‐2‐glycolate moiety and a hydrophobic moiety at the second D‐chiral carbon atom. LDHD has activity towards both D‐lactate and the mentioned D‐2‐hydroxyacids. Additionally, LDHD does not convert the corresponding L‐isomers nor D‐2‐hydroxyacids with hydrophilic moieties [1].

The clinical findings observed in patients with LDHD deficiency occur in a broad phenotypic spectrum, with some patients having only increased plasma urate/gout and others presenting with neurological findings and early‐onset psycho‐motor developmental delay. Delayed motor and mental development were seen in about half of the patients in the literature, corresponding to the findings in our patient and perhaps correlating with elevated urinary levels of D‐2‐hydroxyacids other than D‐lactic acid [2, 4, 13]. In contrast, for patients presenting with early‐onset gout, elevated plasma urate, and without neuro‐cognitive findings, elevated levels of D‐2‐hydroxyacids other than D‐lactate were not mentioned in the literature but may not have been analyzed in this setting [11, 12]. Other clinical features like cryptorchidism, blindness, transient hepatomegaly, and dysmorphic features vary among the patients presenting with primary neuro‐cognitive symptoms [2, 4]. The true fraction of patients with neuro‐developmental signs is uncertain because of the co‐occurrence of other diseases with probable neurological impact in three patients from this group, including 11p syndrome, decreased activity of complex IV of the respiratory chain, and West syndrome [2, 4]. Therefore, in these patients, it is difficult to determine whether the clinical picture can solely be attributed to the LDHD deficiency. It should be emphasized that in the patient with West syndrome, a heterozygous *CACNA1B* variant was found and considered a cause of West syndrome, though this genotype is not a classical cause for West syndrome [2]. In our patient, we also found a homozygous deletion of two genes, *ZFP1* and *CTRB2*, for which there is no known disease association. We found no other genetic variants at WGS, and though it cannot formally be ruled out that *ZFP1* and *CTRB2* contribute to the patient's phenotype, we find it likely that the metabolic findings and his neurodevelopmental symptoms are due to the LDHD defect.

The variants described in the literature in patients diagnosed with human D‐lactate dehydrogenase deficiency vary [2, 4, 11, 12]. Four of the eight different variants identified in the *LDHD* gene were missense variants. Two of these missense variants were identified in patients presenting with gout [11, 12], while the two other missense variants were found in patients with primarily neurocognitive symptoms [2]. Likewise, nonsense variants may be seen in patients from both groups. Altogether, there is no obvious correlation between the type of pathogenic variant of the *LDHD* gene and the phenotype.

## 5 Conclusion

In conclusion, we present a young boy with psycho‐motor developmental delay and metabolic acidosis, who was found to have human D‐lactate dehydrogenase deficiency. Our patient presented with slightly elevated plasma urate, elevated urinary excretion of 2‐hydroxyacids, very increased D‐lactate in plasma and urine, and a homozygous deletion of the *LDHD* gene. Other patients with human D‐lactate dehydrogenase deficiency also present with elevated levels of plasma D‐lactate and increased excretion of D‐lactate in urine—typically in the context of metabolic acidosis with normal p‐L‐lactate and increased urinary excretion of lactate. Our patient presented with psycho‐motor developmental delay, but the phenotypic spectrum is large, with some patients having only increased plasma urate/gout and others neurological findings and early onset psycho‐motor developmental delay; occurrence of urinary D‐2‐hydroxyacids other than D‐lactic acid has only been described in patients with psycho‐motor developmental delay and may be suggested to play a role in abnormal brain development.

## Author Contributions

**T. B. Sloth:** conceptualization, methodology, project administration, visualization, writing – original draft, writing – review and editing. **A. M. Lund:** conceptualization, methodology, project administration, resources, supervision, validation, writing – review and editing. **F. Wibrand:** investigation, methodology, resources, writing – review and editing. **I. Bache:** investigation, methodology, resources, writing – review and editing. **M. C. Ørngreen:** writing – review and editing. **J. Ek:** investigation, methodology, resources, writing – review and editing.

## Ethics Statement

Ethics approval was not required for this study.

## Consent

The family gave consent before any data were collected. Some data cannot be presented here because of the person's sensitive nature of the data, but may be detailed upon reasonable request.

## Conflicts of Interest

The authors declare no conflicts of interest.

## Data Availability

The data that support the findings of this study are available from the corresponding author upon reasonable request.
